# Teachers’ conceptions of learning and teaching in student-centred medical curricula: the impact of context and personal characteristics

**DOI:** 10.1186/s12909-016-0767-1

**Published:** 2016-09-21

**Authors:** Johanna C. G. Jacobs, Scheltus J. van Luijk, Cees P. M. van der Vleuten, Rashmi A. Kusurkar, Gerda Croiset, Fedde Scheele

**Affiliations:** 1 VUmc School of Medical Sciences , Amsterdam & LEARN! Faculty of Psychology and Education, VU University, Amsterdam, The Netherlands; 2 Maastricht University Medical Center +, Maastricht, The Netherlands; 3 Maastricht University, Maastricht, The Netherlands; 4 VUmc School of Medical Sciences, VU University Medical Center & LEARN! Faculty of Psychology and Education, VU University, Amsterdam, The Netherlands; 5 VU University and OLVG-West Hospital, Amsterdam, The Netherlands

**Keywords:** Conceptions of learning and teaching, Faculty development, Personal characteristics, Student-centred curricula, Teaching context

## Abstract

**Background:**

Gibbs and Coffey (2004) have reported that teaching practices are influenced by teachers’ conceptions of learning and teaching. In our previous research we found significant differences between teachers’ conceptions in two medical schools with student-centred education. Medical school was the most important predictor, next to discipline, gender and teaching experience. Our research questions for the current study are (1) which specific elements of medical school explain the effect of medical school on teachers' conceptions of learning and teaching? How? and (2) which contextual and personal characteristics are related to conceptions of learning and teaching? How?

**Methods:**

Individual interviews were conducted with 13 teachers of the undergraduate curricula in two medical schools. Previously their conceptions of learning and teaching were assessed with the COLT questionnaire. We investigated the meanings they attached to context and personal characteristics, in relation to their conceptions of learning and teaching. We used a template analysis.

**Results:**

Large individual differences existed between teachers. Characteristics mentioned at the medical school and curriculum level were ‘curriculum tradition’, ‘support by educational department’ and ‘management and finances’. Other contextual characteristics were ‘leadership style’ at all levels but especially of department chairs, ‘affordances and support’, ‘support and relatedness’, and ‘students’ characteristics’. Personal characteristics were ‘agency’, ‘experience with PBL (as a student or a teacher)’,’personal development’, ‘motivation and work engagement’and ‘high content expertise’.

**Conclusion:**

Several context and personal characteristics associated with teachers’ conceptions were identified, enabling a broader view on faculty development with attention for these characteristics, next to teaching skills.

**Electronic supplementary material:**

The online version of this article (doi:10.1186/s12909-016-0767-1) contains supplementary material, which is available to authorized users.

## Background

Teaching approaches of teachers affect students’ learning approaches and thus students’ academic performance [1]. Trigwell and co-workers found that in classes where teachers described their approach to teaching as transmitting knowledge, students were more likely to report surface learning approaches [2]. Deep learning in students, instead of memorizing and reproducing knowledge, is promoted in student-centred curricula like problem-based learning (PBL). According to Harden et al. ‘in student-centred education students have more responsibility towards their own learning (what and how) [3]. In teacher-centred education learning is more passive; the teacher is the key figure, and there is an emphasis on lectures and laboratory work’.

How teachers teach is partly and unconsciously influenced by their conceptions of learning and teaching [2, 4–6]. Possibly, using the parallel between teaching behaviour and health behaviour in general, health behaviour theories can provide some theoretical insights. Cilliers, Schuwirth and Van der Vleuten discuss several health behaviour theories in relation to educational research and come to an eclectic approach model that indicates how health behaviour is affected by contextual and personal factors [7].

In an earlier study, we used the COLT questionnaire to measure teachers’ conceptions of learning and teaching in two student-centered medical curricula [8]. A cluster analysis resulted in five teacher profiles, ranging from a traditional profile aiming to transmit knowledge to a more modern profile aligned with student-centered education [9].

We also found in a multiple regression study that the most important predictor of teachers’ conceptions of learning and teaching was the context factor medical school, next to discipline, gender and teaching experience. However, it remains unclear which specific elements in medical school account for this predictive effect. Moreover, 82 % of the variance in conceptions in this study remained unexplained [10].

In general higher education, studies on teachers and teachers’ behaviour, usually conducted in traditional lecture-based curricula, underlined the impact of teaching environment on teachers’ behaviour [11]. Further, department chairs had a substantial influence through their leadership and management style, [12–14] for example how they handled the tension between research and education [15]. Also, the educational climate in departments and organizations appeared to be of influence [16, 17]. Other contextual factors were curriculum type, governance of the curriculum, department conflicts of agency and structure, and power relations [18].

Important personal factors were teachers’ autonomy and their perceptions of control over what and how to teach [11, 19]. Moreover, work engagement [20, 21] and satisfaction and emotions [22] modified the perception of the teaching environment by teachers.

### Research questions

To our knowledge, no study has investigated the relation between teaching context characteristics, personal characteristics and teachers’ conceptions of learning and teaching, in student-centred medical curricula. We think that more insight into these factors will yield a broader view on how teaching behaviour can be improved, with additional interventions next to faculty development. Our research questions are (1) Which specific elements of medical school explain the effect of medical school on teachers’ conceptions of learning and teaching? How? and (2) Which contextual and personal characteristics are related to conceptions of learning and teaching? How?

## Methods

### Participants

Participants were purposively sampled from participants of a previous study, who had completed the Conceptions of Learning and Teaching (COLT) questionnaire. The COLT questionnaire consists of the three scales: Teacher-centredness, Appreciation of Active Learning and Orientation to Professional Practice [8]. Based on the results of the questionnaire, we selected teachers from two universities for individual interviews. To obtain maximum contrasts we sampled teachers with high and low scores on Teacher-centredness and Appreciation of Active Learning (see Table 1). The participants were also chosen to represent several disciplines and teaching roles as small group teacher, lecturer and coordinator. A small group teacher guides a group of 12 students who work on assignments, in two sessions per week. A lecturer gives lectures to a large group of about 250 students and a coordinator is responsible for the coordination of an integrated course that takes 4 to 6 weeks. Usually a teacher can indicate a preference for a teaching role to his department head, following which teaching roles are divided in close consultation within a department.

**Table 1:** Descriptives of the participants (*N* = 13)

Discipline	Basic sciences: 6
	Clinical sciences: 7
Age	Range: 31–61, Mean 44.7
Gender	Female: 4
Teacher profile	Transmitters (traditional profile): 5
	Conceptual Change Agents: 6 / Facilitators: 2 (more modern profiles)
Medical school	Amsterdam: *N* = 7, Maastricht: *N* = 6
Teaching experience	Range: 5–35, Mean 14.1

### Setting

We conducted our study in two Dutch medical schools with student-centred curricula: Maastricht University Medical Centre, Maastricht (MUMC+) and VU University Medical Centre, Amsterdam (VUmc). Maastricht has a long-standing tradition in student-centred education, problem based learning (PBL) approach, ever since the founding of the university in 1974. VUmc Amsterdam replaced a classical teacher-centred curriculum with a student-centred curriculum as recently as 2005. This difference in tradition is resembled in a difference in teachers’ conceptions of learning and teaching, as well as their corresponding teachers’ profiles. In Maastricht teachers hold less teacher-centred conceptions than in Amsterdam.

Overall the two medical schools were comparable in the distribution of disciplines in the first 3 years of the undergraduate curricula in Medicine. The estimated distribution was about 40 % clinicians and general practitioners, 30 % basic scientists and 30 % other disciplines (psychologists, sociologists).

In both medical schools, groups of 12 students work on assignments in two small group learning sessions each week. Teachers are expected to guide students’ learning activities instead of transmitting knowledge [23]. Further, both curricula offer lectures and practicals next to small group learning. Courses in basic sciences and clinical sciences are integrated in both medical schools.

### Design

As not much is known yet in the literature about the relation between contextual and personal factors that might influence teachers’ conceptions of learning and teaching, we used an exploratory study design. Because we were interested in individual experiences and not primarily in a group experience of a phenomenon, we decided not to use a phenomenographic approach [24]. We preferred an exploratory study design over ethnography as we were interested in the meanings that individual teachers attached to teaching context and personal characteristics, in relation to their conceptions of learning and teaching, rather than in their actions [25]. It was not our intent to reveal one absolute truth for all teachers, but to explore individual meanings. This is in line with a constructivist paradigm. Constructivism emphasizes that there is no absolute truth shared by all and that ‘the reality we perceive is constructed by our social, historical, and individual contexts’ ([26], p. 405).

### Procedure

Prior to the individual semi-structured interviews, the participants received some background information about our first study on teachers’ conceptions of learning and teaching, including the differences we found in teachers’ conceptions between the two participating medical schools. Also, two main questions of the interview were given beforehand: “How do you explain the differences in teachers’ conceptions of learning and teaching between the two medical schools?” and “How did your conceptions of learning and teaching change during your career?”.

During the interviews we intentionally did not discuss the teachers’ scores on the COLT questionnaire measuring their conceptions of learning and teaching, to maintain a safe atmosphere. Instead, we asked probing open-ended questions to collect information about teaching context (medical school, department and educational context) and personal characteristics. The questions were based on previous studies in general higher education, conducted by Prosser and Trigwell [11], O’Sullivan and Irby [27], Norton et al. [28], and Postareff and Lindblom-Ylänne [22].

All the interviews were conducted by the first researcher (JCGJ) between November 2011 and November 2012 and each interview lasted approximately one hour. We adjusted the interview guide in an iterative processs to improve the data collection (see Additional file 1). We continued interviewing teachers until theoretical saturation was obtained. The interviews were audiotaped, transcribed verbatim and summarized for member checking. All interviewees gave their consent and two interviewees added some information, for example, one interviewee gave a more detailed description of the educational meetings he participated in and of how he perceived the collaboration in these meetings.

### Analysis

All coding was done using qualitative data analysis software (Atlas-ti, version 7.5.1). We analyzed the data using a template analysis method [29–31]. This method stimulates researchers to be explicit beforehand about their assumptions. A template is a list of codes which reflect themes and relationships between themes as conceived by the researcher, building on previous research. Our initial template was based on the information we collected in general higher education, about the influence of contextual and personal characteristics on teaching behaviour. This initial template was the starting point for our analysis and the template was modified by iteratively adding, deleting and reorganising themes as coding continued after every interview. The first two interviews were coded in close collaboration by two researchers (JCGJ, VUmc Amsterdam en SJvL, Maastricht) to achieve consensus on the template, the level of detail and modifications in the template. Next the third and fourth interview were coded individually by these two researchers and discussed afterwards. This resulted in consensus about the codes used. Afterwards JCGJ coded the remaining interviews and modified the template in the subsequent iterative proces of data collection and analysis. After the coding, Atlas-ti provided a sorting of quotations, which were summarized in Excel sheets. The full research team discussed the final template and the summary of quotations.

### Reflexivity

The daily research team consisted of JCGJ, a medical doctor and an educationalist and SJvL, who is a senior staff member trained in medicine with a wide experience in the Maastricht medical curriculum. They were educated in a teacher-centred medical curriculum and involved in the implementation of the student-centred curriculum in Amsterdam. JCGJ was also involved in the training of teachers for small group education during which the impact of teachers’ conceptions of learning and teaching gained her attention more and more. Before the first interview was conducted, the interview guide was elaborated on thoroughly by JCGJ and SJvL. This stimulated us to be explicit about our assumptions. Afterwards, in the coding process, we discussed the first two interview transcripts extensively including the initial template. The richness of the interviews introduced a broad scope on individual teachers’ conceptions. We aimed to elicit implicit assumptions and socialisation effects by the close collaboration of these researchers in the coding process. In the second part of the study, the daily research team was joined by another senior researcher, RAK, also trained in medicine with a PhD degree in medical education. The supervising team consisted of one professor of medical education who is also a gynecologist, one professor of medical education who is a medical doctor and program director of an undergraduate curriculum in Medicine, and a professor of medical education who is a psychologist. The members of the research team work both in VUmc Amsterdam and in Maastricht University. They promote student-centred teaching in their universities.

We are aware that we started the study with the assumption that teachers’ conceptions of learning and teaching change over time. This assumption is strongly grounded in the literature, but the results should be interpreted keeping in mind the researchers’ position on this topic.

## Results

In total we invited 16 teachers for an interview, three of them declined to participate because of time constraints. Two worked at Maastricht University (both from clinical sciences, facilitator profile), and one worked at VUmc Amsterdam (a basic scientist, transmitter profile). We had no indication that the participating teachers differed from the three teachers who declined to participate. The characteristics of the interviewees are presented in Table 1. After 13 interviews theoretical saturation was obtained.

Below we present our results categorised into context and personal characteristics. At the end of the Results section, we will answer our research questions.

### Teaching context

In line with previous studies in higher education, aimed at teachers’ conceptions and approaches to teaching, we identified several characteristics at different levels in the teaching context. We describe our findings at the level of medical school and curriculum, department and educational setting.

#### Medical school and curriculum

Most teachers thought that the overall difference in conceptions of teachers between the two medical schools, originated from the different tradition in PBL and the direction set out by the educational department. Also, they said that the major curriculum change from a traditional lecture-based curriculum to a student-centred curriculum required a concomitant change in teachers’ conceptions of learning and teaching, which needs time.

Irrespective of their conceptions of learning and teaching, teachers expected the Dean and the Director of the medical education program to advocate education at the level of the university and the university medical center, and to facilitate it by earmarking finances, personnel, and time. In both medical schools the financial rewards provided by the educational department for the delivery of education were an important source of income for many departments.

Some teachers were critical about a directive management style and preferred a more supportive role for the educational department, instead of a leading adminstrative position. Teachers rather than the educational department should be viewed as ‘the owners of the curriculum’. Furthermore, they would like the tasks and responsibilities of the educational department to be limited to practical issues, such as grades registration and time tabling. Some teachers in the medical school who recently implemented the student-centred curriculum felt restricted in their autonomy to choose what and how to teach, not only by the leadership style but also by the student-centred curriculum. They thought the student-centred curriculum was more restrictive than the former, lecture-based curriculum (See Table 2).

**Table 2:** Context characteristics: Medical school and curriculum - quotations

	*Teachers with high scores on Teacher-centredness and low scores on ‘Appreciation of Active Learning’*	*Teachers with low scores on Teacher-centredness and high or intermediate scores on ‘Appreciation of Active Learning’*
Tradition; Leadership Dean or Program Director		‘From the beginning there have been discussions with all teachers and students about our view on education, and how best to promote that view‘(M-6)
Major curriculum change		‘I now have to, you know, deliver, I have to talk about this specific topic because it is the central topic in this particular week, and that is, I did not have to do that in the past. And because I almost always invite patients for my lectures, I almost never lecture without patients,..so of course I have to work with the patients who are available. It takes a lot of extra effort to arrange all that, and it makes it all less spontaneous.’ (A-4)
Support educational department	‘For many things you don’t need the support of the Educational Department, in case of a lecture for example, or other parts of a course. But for the construction and logistics of a complete course, and uh… computers, time tables, and the analyses of examinations, all that is supported by the Educational Department..’(M-1)	‘but of course, that will be taken care of by the Educational Department, …our own department facilitates everything for us….and I think there won’t be any problems on that level – or might be….they are very accessible, also in case of problems …’ (M-3)
Management and finances		‘But also, we have to tutor small group learning, for example, …that is very, very, very …..uh, we really have to do that because of the financial rewards for our department’ (A-7)

These quotations from the interviews indicate the association between teachers’ conceptions of learning and teaching and teaching context, at the level of medical school and curriculum

(Coding of respondents: M or A indicates medical school in Maastricht or VUmc Amsterdam; 1 to 7 indicates the number in the respective medical school.

#### Department

The leadership style of department chairs including their vision on education was important for teachers’ conceptions of learning and teaching. Chairs stimulated or frustrated their teachers’ efforts for education because they decided if time or opportunities for faculty development were afforded. However, in some departments the opportunities were limited due to financial constraints. Overall, less time and less affordances were associated with higher teacher-centredness and a lack in teachers’ self-confidence and competence in guiding students in small group education. Clear guidelines in a department as ‘overall, everybody participates in education for 30 % of his job’ were appreciated by teachers, though not clearly associated with high or low teacher-centred conceptions.

Next, the perceived support in their department was also important. With respect to relatedness with colleagues, both teachers with high and low teacher-centred conceptions perceived support from colleagues. Educational meetings on education stimulated this relatedness and support. However, the discussions with colleagues focused mainly on the teaching content and only incidentally on teaching approaches or perceived difficulties in the delivery of education (See Table 3).

**Table 3:** Context: Department - quotations

	*Teachers with high scores on Teacher-centredness and low scores on ‘Appreciation of Active Learning’*	*Teachers with low scores on Teacher-centredness and high or intermediate scores on ‘Appreciation of Active Learning’*
Leadership department chairs	‘In our department it is not as straigthforward as that, because by providing education … for our department, education is an important reason for its existence …. So, providing education is in fact appreciated, but besides that it is of course also appreciated very much if I have a good scientific output.’ (A-6)	‘Department chairs who are completely dedicated to education, yes…, that is not always possible of course, but it is extremely important to involve and engage the other members of the department. Yes, it is very important actually. In fact, I am not sure whether people are sufficiently aware of this. (A-2)
Affordances and support	‘If I know that one of my colleagues developed a course on lungs and I am to give …uh… a lecture on that part of the lungs, of course I contact him and discuss what he did, so I can elaborate on that….’ (M-1)	‘(about department chairs)… they decide if time is allowed to staff members for teaching. They decide who may follow a course … or whether someone gets the opportunity to distinguish himself..’ (A-2)

These quotations from the interviews indicate the association between teachers’ conceptions of learning and teaching and teaching context, more specifically at the department level

(Coding of respondents: M or A indicates medical school in Maastricht or VUmc Amsterdam; 1 to 7 indicates the number in the respective medical school.)

#### Educational context

The respondents indicated that differences in the leadership styles of coordinators were associated with different conceptions of learning and teaching of teachers. For example, if a coordinator inspired students and was respected by them, this positively influenced all the teachers involved in that course. A collaborative leadership style was highly appreciated.

Teachers collaborated across disciplines, sometimes even more than within their own disciplines. Relatedness with peer teachers, the feeling to be connected in a joint effort, was mentioned by the teachers with low teacher-centred conceptions. This teamwork created space for discussion about the success of the course, the perceived difficulties and possible improvements.

Further, teachers’ views on students’ characteristics differed remarkably, in line with their conceptions of learning and teaching. Teachers with low teacher-centred conceptions thought that students were working quite well, and some were impressed with how well students could explain difficult subject matter to peers. Teachers with high teacher-centred conceptions expressed their doubts about the knowledge acquisition by students in a student-centred curriculum. Besides, irrespective of their teachers’ conceptions of learning and teaching, it was said that some students prefer teacher-centred education with knowledge transmission because they want to study strategically and pass their examination with a minimum effort (See Table 4).

**Table 4:** Context: Educational context – quotations

	*Teachers with high scores on Teacher-centredness and low scores on ‘Appreciation of Active Learning’*	*Teachers with low scores on Teacher-centredness and high or intermediate scores on ‘Appreciation of Active Learning’*
Leadership		‘I do not actually feel that I am am being told what to do by B….. as coordinators we try, together with a group of other people who advise us, to develop a certain view on education which on the one hand is not restrictive but on the other hand does not lead to chaos.’ (A-4)
Support and relatedness	‘(during the course…) we have a daily coaching session from ten thirty to eleven o’clock in the morning. During these sessions we discuss the entire course program of that week, you know, including for example…. How did the lecture on heart failure go….’(M-4)	‘.. In teamwork, so that you don’t feel that you are on your own. I mean the peer teachers, as wel as the semester coordinator, and the Educational Department. That there is a team spirit, the sense that we are going to achieve this together. ‘(A-2)
Students’ characteristics	‘However, there are some students that give us the impression that they understand it all, they also work well, methodically including pathophysiology and analysis and differential diagnosis, … whereas other students –and that is difficult to assess during the course […]- that they missed certain connections, and that they only understood bits and pieces.’ (M-4)	‘And that students dare to admit.., well …actually I don’t understand this, can you explain it once again? Some students are really great at explaining, they explain things very well, briefly and concisely, without using difficult expressions.’ (A-3)

These quotations from the interviews indicate the association between teachers’ conceptions of learning and teaching and teaching context, more specifically the educational context.

(Coding of respondents: M or A indicates medical school in Maastricht or VUmc Amsterdam; 1 to 7 indicates the number in the respective medical school.)

### Personal characteristics

There were large differences between teachers in the meanings they attached to personal factors in relation to their conceptions of learning and teaching. Personal experiences as a student and subsequent choices and experiences in their career resulted in a range of professional identities, which combined roles such as teacher, scientist and/or clinician.

Some teachers with high teacher-centred conceptions explicitly reported agency, and said that they wanted to have a direct influence on the content and the structure of the curriculum instead of just guiding small group education. The combination of delivering education and coordinating and/or developing courses was perceived as valuable, challenging and interesting. They found it difficult to change their teaching strategies towards student-centred teaching and preferred to share their content expertise with students instead of guiding and scaffolding their learning. Teaching in small groups, which was not compatible with their high teacher-centred conceptions, decreased their motivation to teach and their work engagement. These teachers with high teacher-centred conceptions were trained in a traditional curriculum. In contrast, experience with PBL as a student or as a beginning teacher seemed to be associated with low teacher-centred conceptions.

Personal development and teaching experience were associated with low teacher-centred conceptions. With increasing teaching experience, self-confidence and feelings of competence increased, and teachers’ conceptions of learning and teaching became less teacher-centred. As beginning teachers, most respondents found it hard to adopt a student-centred learning format and to give students the responsiblity for learning. Their primary concerns were to master the content themselves, to be able to answer all possible questions and to deal with uncertainty and nervousness. Some teachers perceived a lack of time to invest in their professional development as teachers, e.g. in long faculty development trajectories (See Table 5).

**Table 5:** Personal characteristics - quotations

	*Teachers with high scores on Teacher-centredness and low scores on ‘Appreciation of Active Learning’*	*Teachers with low scores on Teacher-centredness and high or intermediate scores on ‘Appreciation of Active Learning’*
Agency	‘if I only were a tutor in various courses, so if I had to fill my educational load that way, that would secriously reduce my motivation, I wouldn’t like that. I really… eh… like having an influence on the content or the composition of a course.’ (M-1)	
Experience with PBL (as student or teacher)		‘I studied in X, a traditional lecture-based, teacher-centred curriculum, so when I started working in Maastricht, that was completely new for me. And when I started to teach it became clear that I didn’t dare to give much freedom to the students, in the beginning.’(A-5)
Personal development	‘ …because traditionally university teachers are hardly trained in didactics; it is taken for granted that our expertise automatically equips us to teach well‘ (A-6)	‘In the beginning I was only focused on my slides and ‘hey, how am I doing?’ Do you know what I mean? And now I think, okay, what are the learning goals, what do I want my students to learn? How am I going do it? How can I arrange this in such a way that they are going to engage with the study material? That is what you have to learn as a teacher. (A-2)
High content expertise	‘I think - you’re obliged to transmit the knowledge you gathered– uh – during your studies and … your working experience… to pass all that on to … say…younger colleagues. That…that is just an intrinsic…., eh yes, that is part of the job.’ (M-4)	
Motivation and work engagement	‘I think - you’re obliged to transmit the knowledge you gathered– uh – during your studies and … your working experience… to pass all that on to … say…younger colleagues. That…that is just an intrinsic…., eh yes, that is part of the job.’ (M-4)	‘Through the interaction…. That is my ….. as a tutor, ….for example I ask them something… then using their answers I ask them another question, and then you see them think hard, and finally and that is what I also like very much, is to see them come up with the answer themselves.’(A-7)

These quotations from the interviews indicate the association between teachers’ conceptions of learning and teaching and personal teacher characteristics

(Coding of respondents: M or A indicates medical school in Maastricht or VUmc Amsterdam; 1 to 7 indicates the number in the respective medical school.)

To answer the first research question, our results revealed several elements which explain the predictive effect of medical school, for example the PBL-tradition, leadership style of Dean, curriculum change. With respect to the second research question, which contextual and personal characteristics are associated with conceptions of learning and teaching, we presented several contextual and personal factors (see Table 6).

**Table 6:** Context and personal characteristics associated with teachers’ conceptions of learning and teaching

Context characteristics	Personal characteristics
Medical school & Curriculum	Agency
• Curriculum tradition	Experience with PBL
• Leadership style Dean / Program Director	Personal development
• Support by Educational Department	High content expertise
• Management and finances	
Department	
• Leadership style department chairs	
• Affordances and support	
Educational context	
• Leadership style coordinators	
• Support and relatedness	
• Students’ characteristics	

## Discussion

This exploratory qualitative study investigated which contextual and personal characteristics influenced teachers’ conceptions of learning and teaching, in the context of student-centred curricula. These curricula promote deep learning of students, by engaging them in active student-centred learning formats [3, 23]. Key elements of problem-based learning which is the student-centred curriculum type in Maastricht, are constructive learning, self-directed learning, collaborative learning and contextual learning [32].

Previously, medical school appeared to be the most important predictor of teachers’ conceptions of learning and teaching, next to discipline, gender and teaching experience. We were interested in the elements of medical school explaining this predictive effect and other contextual and personal characteristics that contribute as well. Analysis of individual interviews with teachers identified several characteristics which were related to low or high teacher-centred conceptions of teachers and high or low appreciation of active learning.

At the level of the teaching context, teachers’ conceptions of learning and teaching appeared to be strongly influenced by the medical school, but also by the leadership style in departments and the educational context. Most teachers thought that the long-standing tradition of PBL in Maastricht was the most important explanation for the differences in teachers’ conceptions of teachers between the two medical schools. However, other effects of organisational culture might have played a role, for example educational climate, appreciation for teaching, a sense of belonging to a community of teachers, or individual socialisation processes.

Teachers differed in their opinions about student characteristics and curriculum type. Teachers with low teacher-centred conceptions thought that students were studying quite well in the student-centred curriculum, whereas teachers with high teacher-centred conceptions had doubts about knowledge acquisition of students in a student-centred curriculum. Prince and co-workers [33] and Schmidt and co-workers [34] found no negative effects on knowledge acquisition in PBL when compared to non-PBL curricula.

The impact of leadership style of the department chairs found in this study is in line with several studies in other domains of higher education (e.g. [12, 13, 15, 35]). However, ‘leadership suffers from a branding problem among trainees, who often equate it with official positions of authority’ ([36], p. 1441), resulting in low participation of department chairs in leadership training courses. Lieff and co-workers explored the needs of department chairs in a faculty of medicine [37]. These needs comprised an increased awareness of the culture and structure of the organization, a comprehensive network of support, interpersonal skills (such as valuing others, conflict management, effective communication) and the ability to influence others. Furthermore, from the perspective of teachers’ conceptions of learning and teaching our study highlights the importance of the vision on education, as well as affordances and support and relatedness with colleagues.

Among the personal factors, the teachers in our study attached great importance to agency. Based on their expertise they prefer to be involved and to participate in the curriculum development [18]. We assume that if teachers are more involved in a discourse about learning and teaching, they might be more inspired and challenged, and possibly develop more student-centred conceptions of learning and teaching. However, the relation might also be reciprocal: teachers with low teacher-centred conceptions may prefer to participate more in other educational fields.

Early experience with PBL as a student or as a beginning teacher, was related to low teacher-centred conceptions. By experiencing the student-centred approach themselves, these participants had naturally developed a student-centred repertoire as teachers. This is in line with Bernstein et al. [38] and Maxwell and Wilkerson [39], who reported that direct experience with problem-based learning resulted in more positive opinions about PBL among students and teachers.

The impact of personal development on teachers and their teaching approaches has been described before by Postareff et al. [6] and Postareff and Lindblom-Ylänne [22] in their studies on the effects of long-term faculty development trajectories. Previously, we established a small predictive effect of teaching experience on teachers’ conceptions of learning and teaching [10].

Motivation and work engagement were high for all teachers, irrespective of high or low teacher-centred conceptions. This emphasizes that next to teachers’ conceptions of learning and teaching other teachers’ characteristics should be taken into account. To implement a student-centred curriculum it is necessary to have sufficient teachers for small group teaching with congruent conceptions of learning and teaching. However, we think that diversity in teachers should be welcomed and will contribute to an inspiring learning environment for students. Students’ learning styles differ as well, and some students will have a preference for teacher-centred education. Moreover, in the two medical schools included in this study weekly lectures are also part of the curriculum.

The interaction with students was highly valued. Van den Berg and co-workers reported as motivating elements teaching about their own speciality, a direct superior with noticeable appreciation for teaching, teaching small groups, feedback on their teaching performance, and freedom to determine what to teach [20]. This is in line with research on work engagement [40] and the Self-Determination Theory [41, 42]. SDT indicates that intrinsic motivation depends on teachers’ feelings of autonomy, competence and relatedness. Possibly, teacher motivation based on autonomy, competence and relatedness might be intermediate variables in the relationship between teacher characteristics, teaching context and teachers’ conceptions of learning and teaching.

To conclude, we have presented some insight in the personal and contextual factors that might be associated with teachers’ conceptions of learning and teaching. A preliminary overview of these factors is presented in Table 6. The combined effect of personal and contextual factors relate to Health Behaviour Theories [7]. From a theoretical perspective on faculty development, choosing a focus on teaching behaviour implies that influencing teachers’ conceptions of learning and teaching towards more student-centred conceptions requires attention to personal and contextual factors as well.

Another theoretical perspective might be the learning process of teachers. Our findings also resemble the 3P (Presage-Process-Product) model which Biggs used to describe student learning [43]. In this model, he combined student factors such as prior knowledge, ability and preferred approaches to learning, and teaching context factors such as objectives, climate and teaching as ‘Presage’. These factors interact with learning activities (‘Process’) and learning outcomes (‘Product’) in the teaching and learning system. Another scope on teacher learning that might fit with our findings, is provided by Illeris [44] who described a comprehensive theory of learning with a cognitive, emotional and social dimension in learning. Illeris stated that this theory might be helpful to promote lifelong learning as well to understand non-learning or resistance. Our findings might contribute to build a new perspective on teachers’ conceptions of learning and teaching in medical education, applying the comprehensive theory of learning described by Illeris for teacher learning and thus acknowledging the impact of personal and contextual elements in faculty development.

### Strengths and limitations

A strength of our study is that we purposively sampled teachers with high and low teacher-centred conceptions of learning and teaching in order to obtain maximum contrasts in our interviews. Another strength is that the coding was performed in close collaboration with a second researcher, based on a discussion of the initial template and its subsequent modifications, and that the interpretation of the results was discussed with the full research team. Finally, we think that we offered insights which can contribute to a broader and theoretical approach of faculty development, by paying attention to the influence of personal factors and teaching context on teachers’ conceptions of learning and teaching.

A limitation of our study is that teachers with intermediate teacher-centred conceptions were not included in our study. However, because of the theoretical saturation we found in our interviews, we suppose that inclusion of teachers with intermediate teacher-centred conceptions would not have enriched our findings. Nevertheless, in a follow-up study we aim to include a broad sample of teachers. Perhaps the teachers with intermediate teacher-centred perspectives will add interesting insights because of their key position in a change process to student-centred education. Also, a bias might have occurred due tot the fact that the interviewees completed the COLT questionnaire before. Also they might have been primed by our previous findings, or their conceptions might have changed over time. Since the study was performed in two medical schools in the Netherlands, follow-up studies are needed to assess the transferability of our findings to other medical schools and other countries.

### Implications for future research

We recommend further research to build on our preliminary overview of personal factors and teaching context factors that are related to teachers’ conceptions of learning and teaching, for example in a large scale quantitative study using structural equation modelling. Also, a longitudinal design study with repeated evaluations of teachers’ conceptions and follow-up interviews aiming at how the teachers perceive their learning process might be interesting. Another suggestion is to explore the effects of a leadership training course for department chairs on the conceptions of learning and teaching of teachers in their departments. Finally, we recommend further research on the interplay between teachers’ conceptions and teachers’ motivation, related to autonomy, competence and relatedness, from the perspective of the Self-Determination Theory.

### Implications for practice

First, our results indicate the importance of the leadership style of department chairs. This might have consequences for the appointment of new department chairs and for leadership courses for department chairs. Secondly, in a curricular reform from a lecture-based curriculum to a student-centred curriculum, ample time needs to be provided for the change process [45], including time for adaptation of the teachers’ conceptions of learning and teaching.

With respect to personal characteristics, we suggest to ask teachers to participate in creating the content and the structure of the curriculum. This might be labelled as ‘participatory design’, which is common in information technology, health promotion activities and in instructional redesign in secondary education [46]. The key point of this approach is that end users of a system participate in the design of that system. We also advise to offer junior teachers first-hand experience of student-centred education early in their career. Further, we suggest to challenge teachers with high teacher-centred conceptions and expose them to student-centred teaching formats, and thus influence their conceptions [38, 39, 47].

## Conclusions

This study identifies several new context and personal characteristics which are associated with teachers’ conceptions of learning and teaching, in student-centred medical education. Especially the impact of the department and the department chair in a medical school was an unexpected outcome. The findings enable a broader view on faculty development with attention for these characteristics, instead of only addressing teaching skills. Further, several theoretical directions to choose in future faculty development initiatives are provided, like choosing a focus on teacher behaviour and using insights from Health Behaviour Theories, or choosing a focus on teacher learning and building on to the 3P (Presage-Process-Product) model by Biggs or the learning theory provided by Illeris.

## Additional file

Additional file 1: Interview guide. (DOCX 18 kb)

## Glossary

Conceptions of learning and teaching: Thoughts or ideas teachers have about learning and teaching. An important feature is that conceptions are partly unconscious and that they influence teachers’ intentions for teaching. Pratt (1992) and Kagan (1992) introduced the definition *‘specific meanings attached to phenomena which can act as a filter through which new information passes as itis processed’.*
References: Pratt DD. Conceptions of teaching. *Adult Educ Quarterly.* 1992;42(4):203–20.: Kagan DM. Implications of research on teacher belief. *Educ Psychol.* 1992;27(1):65–90.
