# Supplementary material for: Teachers’ conceptions of learning and teaching in student-centred medical curricula: the impact of context and personal characteristics
Source: BMC Med Educ. 2016 Sep 21;16:244. doi: 10.1186/s12909-016-0767-1 (PMC5031323; doi:10.1186/s12909-016-0767-1)
Supplement: Additional file 1: — Interview guide. (DOCX 18 kb) [file 12909_2016_767_MOESM1_ESM.docx]

**Interview Guide**

| **Main questions** |  |
| --- | --- |
| **Q1:** | How do you explain the difference in teachers’ conceptions of learning and teaching between the two medical schools? |
| **Q2:** | How did your conceptions of learning and teaching change during your teaching career? |
| **Probing questions** |  |
| Which subject do you teach? | How long? (in this medical school or elsewhere?) |
|  | What type of task in education? Also research and/or patient care? |
|  | Acquainted with student-centred learning, before start as teacher? |
|  | Preparation for teaching? |
|  | Was the PBL curriculum part of the motivation to apply for a job here (in Maastricht)? |
| About the curriculum | General opinion on your curriculum? |
|  | How do you value the integration of disciplines? |
|  | Or competency-based education? |
| Students | Can you estimate what students know? |
|  | Do students appreciate it when you explain difficult material? |
| Appreciation of education in medical school (macro level) |  |
| In your department (meso level) | Opinion about leadership style of department head?  Are tenure tracks in education possible?  Support or relatedness of colleagues, regarding education? |
|  | Are there regular meetings dedicated to education? |
|  | Are all colleagues involved in education?  Do teachers have a choice in type of education they offer? |
| In educational context (meso level) | What is your opinion about the leadership of the (course) coordinator? |
| Personal questions | Autonomy? |
|  | Involvement in education: type? hrs? |
|  | Feasibility? |
| Support by Education Dept. |  |
| Age |  |
| Efficacy and capability as a teacher (yes-no statements) | I think.. that I have the required skills to teach this course;  …that students will learn from me in this course;  …that I master the knowledge needed for this course |
| Are you motivated for your educational activities? | What influences your motivation? |
| Are your colleagues in general enthousiastic about education? | What influences your colleagues’ enthousiasm? |
| How do you perceive the contact with students? | Stimulating? |
|  | What is your opinion about small group learning? About coaching students? |
